# Mixed methods study of a new model of care for chronic disease: co-design and sustainable implementation of group consultations into clinical practice

**DOI:** 10.1093/rap/rkaa003

**Published:** 2020-01-28

**Authors:** Michele Russell-Westhead, Nicola O’Brien, Iain Goff, Elizabeth Coulson, Jess Pape, Fraser Birrell

**Affiliations:** r1 Medical Research Council Versus Arthritis Centre for Integrated Research into Musculoskeletal Ageing (CIMA), Newcastle University, Northumberland, UK; r2 Northumbria University, Newcastle upon Tyne, Northumberland, UK; r3 Rheumatology, Northumbria Healthcare NHS Foundation Trust, Northumberland, UK

**Keywords:** quality of health care, chronic disease, pain assessment and management, patients, outcomes, group consultations

## Abstract

**Objectives:**

Group consultations are used for chronic conditions, such as inflammatory arthritis, but evidence of efficacy for treatment to target or achieving tight control is lacking. Our aim was to establish whether group consultation is a sustainable, co-designed routine care option and to explore factors supporting spread.

**Methods:**

The study used mixed methods, observational process/outcome data, plus qualitative exploration of enabling themes. It was set in two community hospitals, in 2008–19, with a third hospital from 2016, and was triangulated with primary care qualitative data. There was a total of 3363 arthritis patient attendances at 183 clinics during 2008–19. The early arthritis cohort comprised 46 patients, followed monthly until the treatment target was achieved, during 2016–19. Focus groups included 15 arthritis and 11 osteoporosis group attendees. Intervention was a 2 h group consultation, attended monthly for early/active disease and annually for stable disease. Measurements included attendance, DAS, satisfaction and enabling themes.

**Results:**

There was a mean number of 18.4 patients per clinic (*n* = 16, 2010–15; *n* = 18, 2016; *n* = 20, 2017; *n* = 23, 2018–19). Forty per cent (1161/2874) of patients with DAS data reached low disease activity (DAS < 3.2) or remission (DAS < 2.6). Forty-six early arthritis patients followed monthly until they achieved remission responded even better: 50% remission; and 89% low disease activity/remission by 6 months. Qualitative analysis derived five main enabling themes (efficiency, empathy, education, engagement and empowerment) and five promotors to translate these themes into practice (prioritization, personalization, participation, personality and pedagogy). Limitations included the prospectively collected observational data and pragmatic design susceptible to bias.

**Conclusion:**

Co-designed group consultations can be sustainable, clinically effective and efficient for monthly review of early active disease and annual review of stable disease. Promoting factors may support effective training for chronic disease group consultations.

**Key messages**

- A group consultation model co-designed with patients delivered excellent patient outcomes and was sustainable at scale.
- A group consultation model can be used to implement group consultations widely in different health-care settings.
- Key enabling factors: efficiency, empathy, education, engagement and empowerment.
- Active ingredients: prioritization, personalization, participation, pedagogy and personality.

## Introduction

Inflammatory arthritis encompasses a group of chronic, painful and disabling conditions, the most common form of which, RA, affects 4.2% of the US adult population [1]. The total cost to the US economy is estimated at $19.3 billion per annum [2]. Early diagnosis and aggressive management have been proved to reduce morbidity and prevent disability. The UK National Institute for Health and Care Excellence (NICE) clinical guidelines for patients with RA, the commonest form of inflammatory arthritis (NG100) [3] are consistent with the ACR guidance [4] for a tight control strategy, with monthly review and treating to target [5] (inspired by diabetes care). These two major concepts have large service implications: regular, preferably monthly, review of patients with poor disease prognosis until the achievement of an agreed treatment target; and annual review of patients who have stable disease. In practice, rheumatologists also manage PsA using the same treatment strategies, and the evidence base to support this is now robust [6, 7]. In the current UK health economic climate, as in other countries around the world, many units lack the clinical capacity to deliver this enhanced workload model to manage inflammatory arthritis; therefore, other models of care need to be considered.

Demand to care for those with long-term conditions is high and set to rise exponentially as the population ages. This is a universal issue worldwide; caregivers are overwhelmed [8]. The current consultation model provides little time to support people in taking control and self-managing their health. Research suggests that people retain 20–60% of the information (forgetting 40–80%) in one-to-one consultations [9]. Furthermore, staff well-being is affected by current practice, with primary care clinicians reporting unprecedented levels of stress and burnout. In hospital settings, the link between burnout and compromised patient safety is well established and likely also to apply in primary care [8]. Under the current consultation model, no one is getting what they need to keep well. Scaling up group consultations, an innovation already established in other countries, could be part of the solution that simultaneously creates time to care and improves patient activation and staff–patient life outcomes [8].

Group consultations are a way of delivering specialist-led care in groups, rather than individual consultations, that generally focus on clinical management and advice, in addition to patient education and peer support. They have the potential for effective health care within these resource constraints, but the approach is not yet widespread outside the USA. First described in 1974 as cluster visits, group consultations have been widely used in the USA, largely for people with long-term conditions, and usually take place in hospital outpatient services but may also feature in community-based clinics or polyclinics. They have been used by a large health maintenance organization, Kaiser Permanente, to see patients in primary care or with a single problem in secondary care. Group consultation models take a variety of forms, such as shared medical appointments, physical shared medical appointments, self-management, drop-in group medical appointments, group visits and co-operative health-care clinics, and this was flagged as one area to benefit from a systems approach to implementation [10]. They have been shown to be more effective than usual care for antenatal care, diabetes and hypertension [10] and for multiple coexistent conditions [11]. There is also some evidence of potential cost savings and improved patient satisfaction with varying degrees of effect in relationship to empowerment, shared experience and increase in knowledge; a study comparing patient group interventions with traditional one-to-one consultations revealed that patients felt they had better access to care and were more satisfied overall with the care that they received in the group clinic setting [12].

If group consultations provide good clinical outcomes, are popular with patients and cost-effective, why are they not routinely used for patients with long-term medical conditions in the overburdened National Health Service (NHS)? The literature argues that extremely complex clinical and organizational settings present substantial barriers to implementation of any health-care innovation, ‘leading to model modification, incomplete implementation, or failure to successfully embed interventions within health care systems’ [13]. A US study of pre-natal group clinics reported bureaucratic organizational structures, lack of buy-in and financial resources, and staff who were overwhelmed by the model’s challenges as reasons why clinics fail [14]. Group clinics are a substantial paradigm shift from individual one-to-one consultant-led care, which has significant resource, operational and cultural implications. With the majority of evidence for group interventions based in the USA and a perceived dearth of evidence elsewhere, it has been difficult to ascertain whether the model works in the NHS and other socialized health-care systems. In order to roll out group consultations, a recent editorial by Ramdas and Darzi [15] provided four reasons for the slow uptake and suggested conditions that need to be present to improve this. Expressed as crucial enablers for group consultations to be embedded as a service delivery standard, these are as follows: collecting rigorous scientific evidence; discovering easy ways to pilot and refine approaches before fully adopting them; regulatory change or incentives supporting the use of such models; and relevant patient and clinician education.

The aim of the present study was to confirm the feasibility and sustainability of the group consultation model as an alternative to one-to-one appointments in the NHS. We demonstrate the application of group clinics in rheumatology, developed and implemented within a 10-year longitudinal, mixed methods study and adapted for a range of settings and long-term conditions.

The objectives were as follows: to articulate the co-design process used in the development and delivery; to demonstrate that key disease outcome data for inflammatory arthritis (as a prototypical chronic condition) can be collected from a group consultation model and that outcomes and efficiency compare favourably with usual care; to identify enabling themes and promoting factors for the success, acceptability and translatability of the model; and to make recommendations for the rolling out and scaling up of group clinics.

The relevance of this paper to the group consultation evidence base is that it articulates the process of co-design of the group clinic model with patients and key stakeholders. The process covers piloting and implementing an idea, becoming an embedded and sustainable model across an entire service, and adapting the model to suit a range of health-care settings and patient populations in an iterative cycle of feedback and refinement. Although this process has undoubtedly been followed in the USA, where, for example, the Cleveland Clinic has embedded group consultations, delivering 100 000 shared medical appointments [16], the present study provides evidence of the generalizability to other countries and socialized health systems. The recent editorial on group consultations [8] provoked intense debate about the importance of patient involvement in development and evaluation [17], and there has been further recent national media attention, making this front-page news. The aim of this study was to contribute evidence that patient co-design facilitates the adoption and sustainability of group clinics in a wide range of long-term conditions in the UK, in addition to informing the international evidence base.

## Methods

### Study design

**Fig. 1:**
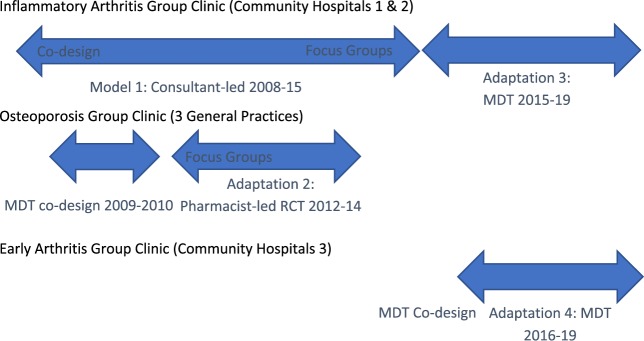
Group clinic co-design timeline (with patients and multidisciplinary team) and delivery MDT: multidisciplinary team.

This is a 10-year longitudinal study detailing the development of a group consultation model at Northumbria Healthcare NHS Foundation Trust. Sustainable models were derived and adapted from an original consultant-led group clinic for both active early and stable inflammatory arthritis, which was subsequently operationalized in a range of settings and chronic conditions (see Fig. 1). We use a mixed methods approach, defined as incorporating both quantitative and qualitative data (in this case, a triangulation multilevel model) to collect key clinical outcome and patient satisfaction data and involved patients and key organizational stakeholders to help co-design, evaluate and refine the model by identifying the key enablers for successful implementation. An adapted experience-based co-design model [18] was adopted, in which patient feedback during clinics helped to inform the clinic design. In health care, the term co-design refers to patients and carers working in partnership with staff to improve services [19]. The clinics were held in a community hospital setting or in general practice.

Observational data (good for capturing process flow, satisfaction and outcomes) were triangulated with observational and in-depth interview data (qualitative; good for exploring complexity, including reasons for success) from staff and patients attending a pharmacy-led osteoporosis group clinic in general practice [20] and patients attending a homogeneous early arthritis multi-professional group clinic. The qualitative data also refined the approach for subsequent clinics.

Ethical approval was granted by Newcastle and North Tyneside 1 Research Ethics Committee (REC approval reference 10/H0906/88).

### Patient involvement

Patient involvement in the group clinic has been integral from the outset and at every stage, including development, delivery and quality improvement (see figure 2 of Jones *et al.* [10]). This has been articulated in a rapid response to an editorial [21], including initial scepticism by the patient representative working with the Rheumatology team when the first pilots were proposed in 2008, which was overcome by the experience of attending in 2017. We hoped to show that with true, iterative co-design, initial concerns may be overcome, and a deep connection with the group clinic model can develop. The patient voice directly informed development the model, especially in response to challenges (see Tables 1 and 2). The results have been used to refine the model and have been disseminated to patients at the group clinics. In addition, patient representatives have been involved actively in contributing to grant submissions and publications.

**Table 1:** Group clinic model adaptations to meet service and patient need

	Osteoporosis group clinic: pharmacist-led clinic	Group clinic: consultant-led MDT clinic	Early arthritis group clinic: consultant-led MDT clinic
Clinic administration	Administration streamlined; available ahead of clinic	Administration streamlined; available ahead of clinic	Administration streamlined; available ahead of clinic
Introduction	Introduction and ground rules (shared confidentiality; balancing contribution)	Introduction and ground rules (shared confidentiality; balancing contribution)	Introduction and ground rules (shared confidentiality; balancing contribution)
Education and tools	FRAX score self-calculated	Created an educational poster (Supplementary Fig. S1, available at *Rheumatology Advances in Practice* online) HAQ self-completed	Educational posters HAQ completed with nurse
Additional staff	None; clinic facilitated by pharmacist Usually led by a single pharmacist, although pilot clinics had a specialist nurse and/or expert consultant present for model development and training	MDT member (specialist nurse, occupational therapist, physiotherapist, podiatrist or pharmacist, depending on availability) delivers arthritis education for 1 h Clinic nurse	MDT member (6-monthly rotation, including specialist nurse, occupational therapist, physiotherapist and podiatrist) delivers arthritis education for 1 h Clinic nurse/health-care assistant
Investigations and procedures	No blood tests required	Clinic nurse organizes clinic and undertakes clinical procedures (e.g. i.m. CS injections and blood tests, if needed)	Clinic nurse organizes clinic and undertakes clinical procedures (e.g. i.m. CS injections and blood tests, if needed)
Location and format	≥10 patients in a general practice	12–32 patients in a group education room in a community hospital	14–20 patients in a group education room in a community hospital
Micro-consultations	No parallel micro-consultations Pharmacist-facilitated group session covering osteoporosis, fracture risk, lifestyle and treatment	Consultant undertakes micro-consultations: DAS, treatment choices and provides information leaflet (1–2 min per patient)	Consultant undertakes parallel micro-consultations: DAS, treatment choices and provides information leaflet (∼4 min per patient)
Interactive education	Question and answer session: engaged in discussion, with an opportunity to discuss confidential issues after the group session	Consultant delivers inflammatory arthritis education based on the concerns highlighted by patients during micro-consultation	Consultant leads open question and answer session after completion of micro- consultations and a break
Prescriptions	Offered prescription for alendronate, calcium/vitamin D_3_. Written information/health promotion about osteoporosis	Prescription for new drugs, confidential concerns and joint injections if required	Prescription for new drugs, confidential concerns and joint injections if required
Target patients	One-off clinic to manage those at risk: invitation to review for those who stop therapy feasible	Early arthritis/flaring patients attend monthly clinic until disease is controlled. Stable patients attend annually	Patients attend monthly clinic until disease is controlled
Feedback	Feedback sought and fed into clinic improvement	Feedback sought and fed into clinic improvement	Feedback sought and fed into clinic improvement

MDT: multidisciplinary team.

**Table 2:** Problem solving with iterative co-design: identifying group clinic challenges and solutions

Problem	Solution
Poor response to anonymous invitations (GC/EAGC/OPGC)	Invitation at time of first clinic appointment/flare
Reticence for serial observed consultations (GC)	Adoption of less threatening problem-oriented discussion and micro-consultations
Patients waiting to book in for clinic (GC)	Book patients in once seated in group clinic
Patients queuing for injections after clinic (GC)	Offer injections during clinic
Patients requesting more education from MDT (GC)	Rotating involvement of team members delivering education concurrent with consultant micro-consultations
Patients missing out on education by having injections/ micro-consultation during education (GC/EAGC)	Recap patients on their return (GC)
	Offer choice of injection after clinic/develop posters for core content (IAGC)
Micro-consultations taking too long and too far from group room (EAGC)	Clearer briefing on ground rules of purpose and alternate use of two rooms, improving flow
Lack of interaction in early clinics (GC/EAGC)	Rearrange seating into a circle to encourage interaction
Falling attendance when one consultant was not seeing new patients; less discussion (EAGC)	Invite follow-up patients to attend (the new patients found this very helpful)
Low attendance when clinic was split to prevent overbooking, reducing the group effect (GC)	One group in larger venue; MDT education; option to depart after micro-consultation

GC: group clinic; EAGC: early arthritis group clinic; MDT: multidisciplinary team; OPGC: osteoporosis group clinic.

### Clinical outcome data: methods, patients, sampling and data analysis

The group consultation model with three distinct adaptations over time is described here, to demonstrate how the model is adaptable to different settings and chronic conditions (see Fig. 1). The original consultant-led group clinic in 2008–2015 (observational and qualitative data; model 1) forms the basis of best practice in group clinics and has been constantly evaluated and improved using the iterative co-design model as described above. The three adaptations are as follows: (1) a pharmacist-led osteoporosis group clinic (OPGC) in primary care (qualitative comparison alongside randomized controlled trial with 12-month follow-up) 2012–2014, following multidisciplinary co-design (2009–2010); (2) a multi-disciplinary supported, consultant-led group clinic 2015–2019 (observational data); and (3) an early arthritis group clinic (EAGC) 2016–2019 (observational data).

The primary clinical outcome data (model 1 and adaptation 3 above) are derived from two community hospitals serviced by a single rheumatology unit, consisting of consecutive group clinics since piloting July–September 2008, with routine implementation in May 2010 through to July 2019 (see Table 1). Early data were derived from a consultant-led model, with later clinics adopting a multi-professional approach in response to patient requests. This is triangulated with data from a multi-disciplinary team EAGC (adaptation 3 above; 46 unique patients attending monthly clinics) from July 2016 to October 2018. Data include attendance (for the whole period), i.m. CS injection (collected since August 2012) and DAS [22] (DAS28; collected since November 2012), with categorization of patients as in remission (DAS28 < 2.6) or with low (DAS28 < 3.2), moderate (DAS28 = 3.2–5.0) or high disease activity (DAS28 ≥ 5.1). Corresponding values for PsA were no swollen and tender joints (remission) and one or two swollen and tender joints (low disease activity).

Patient feedback on the content and structure of the clinic was collected at the end of each session using a co-designed, Trust-developed Rheumatology patient experience survey, and the same survey was sent to 437 usual clinic patients, which was completed by 263 patients (60% response rate) for comparison. Similar feedback was obtained from the EAGC in the set-up phase (data not shown). Many group clinic sessions were observed informally and two sessions formally by a clinical education expert, who also undertook the qualitative evaluation (see qualitative evaluation paragraph below), to articulate the clinic structure properly, assess patient engagement in the group situation and identify good practice. These findings formed an educational model to support staff training and development of the approach.

Qualitative themes were triangulated with group clinic feedback and both types of data from a pharmacy-led osteoporosis clinic model (OPGC, variation 1 above; 75 patients and four clinics).

### Qualitative evaluation: methods, patients, sampling and data analysis

A pragmatic qualitative approach [23] was used to explore the experience of patients and ascertain barriers and enablers to the spread of group consultations. Focus group interviews were carried out between June and August 2014 at two hospital sites by two researchers. Researcher A (M.R.-W.) is an educationalist with a clinical health-care, medical education (including doctorate) and policy background, and researcher B (F.R.) is a rheumatology registrar. This mix of disciplinary, professional and research backgrounds was specifically chosen to capture the nuances of the health-care and education models used and the social science aspects of patient experience. The participants were unknown to both researchers.

#### Sampling

Fifteen group clinic attendees self-selected to be interviewed after information about the research project was sent out to all patients invited by mail to the clinics. This was 62% of all patients seen in those sessions. Reasons provided for non-participation in the study were mainly time related. Fourteen were patients (12 women and 2 men) and one was a male relative (Table 3). Informed consent was gained from the patients by researcher B, and they were advised how the data would be used and that they could withdraw from the study at any point and that this would affect their care in no way. All had attended previous one-to-one clinics and at least one group clinic. The focus groups took place immediately after the group clinic and lasted an average of 1 h.

**Table 3:** Patient focus interviewees: inflammatory arthritis group clinics

Hospital A	Hospital B
AA White female 70–79 years old	BA White female 80–89 years old
AB White male 70–79 years old	BB White female 60–69 years old
AC White male 60–69 years old	BC White female 70–79 years old
AD White female 50–59 years old	BD White female 60–69 years old
AE White female 50–59 years old	BE White female 60–69 years old
AF White female 60–69 years old	BFWhite male
AG White female 50–59 years old	(BE’s husband, not RA patient himself)
AH White female 70–79 years old	BG White female 70–79 years old

#### Data collection

Researcher A facilitated the focus group using a semi-structured interview guide, with introductory remarks about the purpose of the study, prompts and some broad opening questions to start the conversation, such as, ‘Tell us about your experience of attending the group clinics’, ‘How does it differ from the one-to-one consultation?’, ‘What do you like about it/what works well for you?’, and ‘Is there anything that could be improved upon?’. Prompts and probes were used by both researchers to elicit rich data from the patients. The interviews were recorded digitally and transcribed verbatim by researcher B, who also made field notes to check findings and act as an aide memoire to the focus group proceedings (for example, non-verbal cues). Transcriptions and interview notes were uploaded into QSR’s NVivo 9 qualitative data analysis software (QSR International, Melbourne).

#### Data analysis

The focus group transcripts were analysed by researcher A using thematic analysis [24] and constant comparison techniques, which are methods widely used for identifying, analysing and reporting patterns (themes) within data. The coding process was started by conceptualizing the meaning given by the patients by reading and re-reading the transcripts. Comments with similar meanings were clustered and give a nominal heading. Coded extracts and sub-codes were then put together and examined. Given that all patients had experience of both group clinics and one-to-one consultations, data were analysed together, and responses for each group were compared within each theme to identify similarities and differences between groups and to develop understandings with practical implications. Data saturation was reached when no new themes were emerging. Both researchers then re-read the data obtained through this system of organization and refined the data by discussing the codes and the themes one by one to reach a consensus on the patient experience of group clinics described by the patients themselves. The results were triangulated with qualitative data from patients in the osteoporosis group clinics [11 white female patients, mean age 70 (range 62–88) years]. In addition, the consultant rheumatologist leading this initiative was also interviewed by researcher B to provide historical insight into the development of group clinics and to examine his perceptions of the differences between the original consultant-led model and the pharmacist-led osteoporosis variant.

This was then written up by researcher A using vignettes from the all the data, the patients’ and consultant’s experiences, to illustrate the themes.

## Results

### Clinical outcome data

From 2010 to 2019, there were 3363 patient attendances over 183 mixed clinics, giving a mean of 18.4 patients attended each 2 h mixed clinic. Mean attendance varied over time, with the pilots in 2008 having 10 (8 and 12) patients; 2010–2015, 15 (range 10–24) patients; 2016, 18 (range 12–26) patients; 2017, 20 (range 14–32) patients; and 2018–19, 23 patients (range 17–28), suggesting that efficiency improved over time, with no reduction in satisfaction (data not shown). Forty per cent (1161/2874) patient attendances with DAS data reached low disease activity or remission. Administration of i.m. CS was more frequent for the later clinics than for the pilots (where 5/20 received it, i.e. 25%): given in 1596 (55%) of 2904 patients where injection data were collected routinely. The more recently established homogeneous early arthritis cohort (EAGC; 46 patients) showed even more impressive response: remission was achieved in 23 out of 46 patients where full data were available (50%) by 6 months, with another 18 patients having low disease activity (39%; giving 89% of the total with low disease activity). Of the five remaining patients who had moderate (*n* = 3) or high disease activity at 6 months (*n* = 2), those who continued in the group clinic reached low disease activity or remission by 8–13 months.

The clinics ran smoothly, with no noticeable waits from arrival for the patients to complete the individual diagnostic/treatment questionnaires and HAQ. Logistically, it was sometimes difficult for the main group clinic lead to complete a DAS28 score on each participant; therefore, this could be delegated to the pharmacy practitioner, rheumatology nurse or trainees, depending on the skill mix available on the day. Patients in the pilots were given the choice of having a serial observed consultation (as recommended in the Kaiser group models), but all groups preferred to raise issues in an initial brain-storming session so that they could be discussed with the group. The topics discussed included the following: aetiology of RA, fatigue, flares (including triggers and management), OA, chronic widespread pain, sleep disturbance, risk *vs* benefits of NSAIDs/coxibs/CSs, exercise, disease-modifying therapy (monotherapy and combination), biologic therapies, impact on work and relationships. A number of challenges were identified and solutions created by the groups (see Table 2). Feedback was very positive (Table 4) across all domains, with no significant differences in satisfaction between the established group clinics (2008–19) and usual care. Satisfaction was equally high for the early pilots (from 2008) and EAGCs (2016–18; data not shown). Even patients who had very severe disease where biologic therapy was contraindicated through infection risk benefitted from the peer support provided by the group clinic.

**Table 4:** Patient evaluation of inflammatory arthritis group clinic and usual care

Question: How would you rate the clinic for… (numerical rating 0–10; where 0=very poor, 10=very good)	Group clinic Median (IQR) *n*=2859^a^ (85% response)	Usual care Median (IQR) *n*=393 (60% response)
Listening to you	10 (10–10)	10 (10–10)
Explaining disease and treatment	10 (9–10)	10 (9–10)
Looking at joints/skin	10 (10–10)	10 (9–10)
Opportunity to discuss treatment options	10 (10–10)	10 (9–10)
Providing treatment	10 (10–10)	10 (8–10)
Access to MDT	10 (10–10)	10 (9–10)

a Total of 3363 attendances; not all patients completed and returned the feedback sheet or answered all questions on it, but the response rate was higher than that for usual clinic care, for a postal questionnaire.

IQR: interquartile range; MDT: multidisciplinary team.

Only 12 patients (0.4%) who completed a feedback questionnaire from all the clinics said that they would not come to further group clinics, and 4 (<0.2%) said they would not recommend them to others. Therefore, even patients who do not personally want to use the model recognize its potential value to others.

### Qualitative findings

The qualitative data analysis from the inflammatory arthritis group clinics identified five main enabling themes (efficiency, empathy, education, engagement and empowerment), which were linked to key indicators of patient satisfaction (see Table 5).

**Table 5:** Enabling themes and promoting factors

Enabling theme	Details	Impact on patient care and satisfaction	Promoting factor and implications for translation
Efficiency	Reduced waiting times More streamlining of administration at clinic More effective use of time; more patients seen in a session Referrals and follow-up	‘It’s very helpful, and if there’s anything that you’re concerned about, it’s easier than waiting for 6 months for an appointment’ ‘You are not waiting in a queue like before; you are straight in and can have tea and a chat while you fill the forms in and wait for the doctor to see you and do your joints’ ‘I think that having a group, obviously more people get seen, which has to be, you know, more effective really’ ‘I always make sure the secretaries follow up people who didn’t come and ask whether they want to be seen in the next group clinic’	Prioritization Translation points: Have buy-in from entire clinical and administrative team and include them in the design, implementation and evaluation of the process Personalization Translation points: Ensuring that there are effective ways of recording events of and action points from the session with individualized follow-up
Empathy	Shared problems Shared understanding Group support	‘We are all in the same boat’ ‘The group understands that the pain gets you down, and it makes me feel better when I hear others describing what I go through every day’ ‘You generally have a little chat while you’re having a cup of tea and can get a little bit of advice about whatever is worrying you’	Participation. Translation points: Need to create sense of belonging and camaraderie
Education	Learning from health-care professional Learning from others	‘I would never know all that about disease, you know if you’re below 3, or you’re below 2.5 you’re in remission…. I’ve got a much better understanding of how my disease works’ ‘I think questions get asked that you might not ask yourself because you might feel silly, so you get the answers that you want’	Pedagogical approach. Translation points: Content matches need, make relevant, provide examples and state what that means to them Participation. Translation points: Collect questions before group discussion Have opportunity for patients to ask questions of each other
Engagement	Appropriate personality, benefits of a trained educator Individualization in a group setting Positive physical and emotional environment	‘I think Dr A is very approachable and he’s got a very good manner and draws people out’ ‘Well he’s very good in that he talks to the group, but also he acknowledges that you’re an individual’ ‘You can go to the other room and get your injection while he is seeing other patients’ ‘You can have a laugh, and it’s more relaxed, and you probably get a bit more out of this than you do from a one to one’	Personality/pedagogic approach. Translation points: Ensure that the right people are leading the session, whio have passion, an interest in teaching and skill Personalization. Translation points: The ability to differentiate in a multi-need group Prioritization. Translation points: Need appropriate premises for delivery and training in facilitation skills for participating clinical staff
Empowerment	Agency, autonomy and advocacy Focus on personal impact Promoting behavioural change and physical well-being	‘I just asked if I could have an injection…. I got one, no problem at all, and I went home feeling on top of the world. You feel like you have some control over your care!’ ‘It’s made me realize I am not that badly off but need to take more control so not to get worse’ ‘I have learnt tips on how to manage my condition better, like doing regular exercise will improve my joints and make me less tired’	Personalization. Translation points: Ensure that the session is made relevant to individual need: specific treatment or advice is available, general topics can be individualized (e.g. use examples from the patients, use names and focus on how knowing this is important and doing this will improve health outcomes) Participation/pedagogic approach. Translation points: Opportunity for both clinician and peer advice and support is most impactful; one validates the other

## Discussion

Rheumatology group consultations can be engaging, empowering and efficient; patients enjoy them and have a sense of involvement in their care, and this study has shown that key disease outcomes can be collected, informing the quality of care delivered. This is achieved by the group clinic model delivering a longer contact time than the one-to-one clinics, thus encouraging more active patient involvement, and has potential to replace some one-to-one clinics. Although not all patients want to try this initially, the present study suggests that a single positive experience can lead to the majority of patients both wanting to use this model of care again and becoming advocates. Given that this group model is a self-replenishing one, there has not been a drop-off in attendance, commitment or enthusiasm over time. This is an important feature to consider, because the studies identified in the evidence review commissioned by the National Institute for Health Research [25] pointed to a reduction of these, particularly in cohort-based clinics, whereby the group clinics studied here have shown that the opposite can be true. Mean attendance at the mixed group clinic increased from 15 to 23. Although most would assume that patient experience would deteriorate when >15 attend, this study provides direct evidence that this is not the case. Indeed, on the rare occasions when numbers were low, there was less discussion. This is a highly counter-intuitive finding, which can inform and change practice. Bigger group clinics can deliver a better experience, as long as the space and staff provision are right, whilst providing greater efficiency.

As can be seen from the qualitative findings, five enabling themes (efficiency, empathy, education, engagement and empowerment) appear to be present in successful clinics, promoting high levels of acceptability and sustainability with this patient population. In concert with this, five promoting factors have been articulated to help put these themes into practice and can broadly be categorized into prioritization, personalization, participation, personality and pedagogical approach.

It is important to provide information ahead of the patient’s first attendance at a group clinic regarding why they have been chosen to attend, the format and what they can do to prepare so that they can get the best out of the clinic and also manage expectations. The way in which these clinics are organized promotes a prompt start, engaging patients immediately on arrival. They afford the opportunity to embed quality/outcome data collection, ensuring evidence-based practice. Education addresses variability, invisibility and makes sense of what is happening. This should ideally include multiple modalities to reinforce learning (e.g. educational poster, Supplementary Fig. S1, available at *Rheumatology Advances in Practice* online). Moreover, group consultations provide one solution to allow delivery of an annual review clinic within constrained resources and to accommodate patients needing monthly review for tight control. Finally, the model is flexible enough to facilitate urgent review for flares or the addition of recently diagnosed patients without the inconvenience for others of an overrunning clinic. This model can be adapted to seeing emergencies; for example, as a drop-in clinic to see patients in primary care at the weekend, or at other times when demand outstrips supply. The total clinic duration (90–120 min) was very similar whether 8 or 32 patients attended, with the logistical limit on group clinic size being the size of room available in interactive layout (usually in the round), in addition to the time taken to carry out joint counts. Carers were welcome to join, as for a normal clinic, but this provided an additional opportunity for their questions and concerns also to be heard and shared without prolonging the session.

There were some differences between the early group clinics and later ones, which is unsurprising because this was part of a co-design process. The main difference in treatments was the increase in i.m. CS injections for the established clinics, compared with pilots. There are several possible explanations for this. The later clinics included the calculation of the DAS28 score; categorization of disease activity as remission, low, moderate or high; articulation of a treatment target (to achieve remission or low disease activity); multi-disciplinary input; and the opportunity to have an i.m. CS injection during the group session, rather than having to wait and stay on afterwards. All of these differences could have increased the proportion of patients choosing to have i.m. CS injection, but as observed in the TICORA study [5], the important observation is probably that more CS injections were given to those with active disease, whatever the element of the care package responsible for this change. A major change in the group clinic model was prompted by the qualitative research as it became clear that patients wanted more education from other team members; therefore, a process to incorporate other team members began. This process was facilitated by the establishment of the early arthritis clinic, with two additional consultants and several other multi-professional team members becoming actively involved, as the culture of the department became much more oriented to group clinic care.

The key strengths of this study are that it is large, long-term, covers multiple settings, clinicians and centres and uses a mixed methods approach. There are some important limitations to what was a pilot service innovation, extended with agreement of the local health-care commissioners. First, this was not a randomized controlled trial, but a mixed methodology study collecting observational data, which was appropriate to meet the objectives stated. There was a direct comparison of satisfaction with usual care using the same tool, but without blinding, the results are potentially more susceptible to bias. That said, blinding of patients is not feasible for this type of package of care study, even in randomized controlled trials, such as TICORA or TICOPA. The proportion of patients achieving low disease activity or remission in the mixed early and chronic disease group clinic is comparable to or better than registry data from two US cohorts, where routine practice includes earlier use of anti-TNF therapy [26]. EAGC data make it clear that the group approach delivers better outcomes; the 6-month remission rate of 50%, and 89% with low disease activity or remission, is equivalent to or better than that achieved in other observational cohorts or controlled trials. TICORA achieved 66% remission after 18 months [5], and TACIT had 34% achieve remission at any time >12 months [27]. The only intervention achieving a comparable remission rate of 55% by 6 months is tocilizumab [28], which has much higher cost. For clarity, by the 6-month time point, no patients in the EAGC had received biologic therapy, although one patient did require biologic therapy to achieve remission at 13 months.

Although the calculation of the DAS28 has been incorporated in the group clinic and is essential in agreeing a treatment target with the patients, this is not collected routinely in one-to-one clinics; therefore, the data from those are not included, although as stated, patients were moved from monthly to annual review on reaching remission (DAS28 < 2.6) or a low disease activity state (DAS28 < 3.2; or fewer than three tender and swollen joints in PsA). Equivalent satisfaction levels in both mixed group clinics and the early arthritis model were seen, compared with the survey of outpatients in the same centres before the group clinics were initiated. However, there was a ceiling effect for the questionnaire used, which would limit the discrimination of any differences. Although satisfaction may change over time, there is no specific reason to believe that the routine service would have changed for the worse over the period of the study, because these clinics were not adversely affected by the group clinics. Indeed, the ability to be able to add urgent extra patients to the group clinics has tended to relieve pressure on the one-to-one clinics, which have been less overbooked as a result, meaning that this delivers benefits even to the patients who do not opt in to this model. In the hospitals where the service is established long term, 40% of follow-up appointments are now delivered through group clinics.

There are two main strands of literature for comparison. The first is that of existing models of care for the management of RA. This includes the predominant model espoused by the EULAR [29], ACR [30] and NICE NG100 [3]. These recommend annual review for stable patients, with the implicit assumption of one-to-one care, but there is little articulation of the resource implications. The UK National Audit Office report [31] suggests that there are significant resource implications to the implementation of a treatment-to-target strategy. By increasing the proportion of patients treated within 3 months of onset from 10 to 20%, NAO estimated a cost of £11 m (€12.3 m or $14.1 m, at 2018 exchange rates) over 5 years, but potentially realizing overall savings to the economy of £31 m (€34.8 m or $39.8 m). Proportionately larger savings could be made in in the USA and other countries, especially if use of expensive biologic therapies can be delayed or avoided.

The group clinic model is capable of delivering many of the components of care that may explain the success of the tight control strategy [5, 6], which was inspired by diabetes care and also applies to other chronic diseases. This includes a large and timely educational component (to maximize early adherence to disease-modifying therapy), regular i.m./IA CSs, rapid supported escalation of disease-modifying drugs and the agreement of a treatment target, shared with both the physician and the patient. Importantly, it provides patients with a greater sense of empowerment and ownership of the management of their disease by providing a social constructivist approach to understanding their condition and learning of a variety of methods of self-help gained from the other patients, which feedback from patients reported to be powerful and comforting (‘if others can manage it, so can I’). The themes we articulate map powerfully to ‘living precariously with rheumatoid arthritis’, from a recent mega-ethnography describing 10 conceptual categories [32]. Engagement and empowerment especially address the control and lack of reciprocity exerted by RA; education addresses the variability and invisibility and makes sense of what is happening. Groups help to reframe the situation and provide a positive experience [32]. Group clinics are also attractive in providing more frequent outcome data than patient-initiated follow-up [33] and a high level of consultant contact compared with the nurse practitioner model [34].

The second strand of literature relates to group clinics in other specialities. As outlined above, the implementation of group clinics for RA differs significantly from the models used by Kaiser, especially in generalizing the problems covered in the clinic, rather than holding a number of individual consultations within a group setting. Indeed, there are at least three distinct models used by Kaiser: the drop-in medical group appointment, shared medical appointment and physical shared medical appointment; hence, comparisons are limited by differing methodologies. However, observational data for a hypertension group intervention, a randomized controlled trial for primary care patients with chronic conditions and secondary care Veterans Affairs Medical Center patients with diabetes and hypertension provide evidence that these approaches can be efficient and cost effective [11]. The National Institute for Health Research-funded systematic review [25] showed that most of the evidence on group clinics is related to diabetes care and practice in the USA. Having shown that it is both feasible and sustainable to implement group clinics in the UK, this justifies their more widespread use in the UK and elsewhere. However, further studies across a range of care settings, chronic conditions and designs, with particular reference to efficacy and cost-effectiveness outcomes, would be helpful.

With regard to implementation, overall, our findings are consistent with previous research studies [14, 35], emphasizing further the need for organizational buy-in, group clinic champions, an investment in training and development of clinical staff in teaching and facilitation and an organization supportive of innovation [10]. Implementing and sustaining the group clinic model is without doubt introducing ‘a new way of thinking, acting or organizing’ [36] into the existing service, which is no small task, and the challenges of both structural and cultural change should not be underestimated. It is important to win over both champions and decision-makers to make it work. In relationship to cost implications, once up and running, group consultations have been proved to be self-sustaining and cost-saving, although this is somewhat determined by local factors when considering economics in the roll out. This study confirms that in the UK, similar efficiency of 200–300% compared with usual care is achievable, as previously shown in the USA [10]. The main strength of this team’s implementation of group clinics was in its experience-based co-design model approach, which held the model to constant critique and refinements based on patient and staff feedback and service need. This also enabled the team to identify factors that were unique to the group clinic model. The open qualitative research design also enabled the identification of the perceived benefits and aspects of the delivery of care that were most important to the participants, rather than what the researchers felt were important.

The qualitative findings could not emphasize more strongly the importance of well-trained (in education and facilitation), knowledgeable and personable clinical staff, and this is supported substantially throughout the literature [10, 12, 14]. It was clear from the patient narratives that it is important that they feel a sense of being treated as an individual in the group setting. This was achieved: before the clinic, in the personalized letter inviting them to the clinic; on arrival, when the staff knew their names and they were dealt with efficiently, not waiting around by the reception window as in the traditional clinics; by the consultant spending time with each of them to talk about their concerns or ask questions; by use of names during the group education session; and by the fact that the consultant remembered their questions and referred back to them. Also, the fact that other members of the group took time to contribute to the discussion on their topic or provided tips for self-management contributed to this sense of individualized care despite the presence of many others in the room. The degree of personalization was largely achieved by the approach to facilitation of the educational component of the clinic adopted. The consultant answered key questions and addressed patients’ concerns but also used a Socratic questioning approach to draw the answers out of other patients. They were encouraged to share their stories, give advice based on their own experience of managing their condition and encouraged to think of the answers to some of the questions posed by others. Some people were more reluctant to contribute but enjoyed listening, and that is acceptable. It needs to be noted, however, that this approach relies on the clinician being skilled in facilitation, having high levels of emotional literacy, the ability to monitor the engagement levels of the individual members of the group and bing able to identify genuine need in the busy and, at times, demanding nature of the clinic. Those patients least confident or able, perhaps through medical or linguistic ability, to communicate and engage in a group setting might be the ones who are most in need of additional individualized care. There is also a need for clinicians to balance explaining in lay language and reference to the evidence base for the information being given. In our study, patients commented on this; for example, the clinician referred to the TICORA [5] study when explaining why it is important to take your medication regularly, and some patients felt this was over their heads, pointing to the need not to become too jargonistic, giving only the key points of that study and what it means for the patient. This provided a supportive, empathetic and proactive environment for patients to have their experience validated and consider their options. The data revealed that this, probably more than anything else, was the key active ingredient of the success of the group clinic.

### Conclusion

This study addresses the issues highlighted by Ramdas and Darzi [15] for any highly innovative service delivery to become standard. First, it is a large mixed methods study showing high patient satisfaction and 39% of patients achieving low disease activity or remission in heterogeneous and 89% in homogeneous EAGCs (50% remission). With a current mean clinic size of 23 patients and 40% of the Rheumatology outpatient delivery workload for the two original hospitals, the key findings of this study are that group clinics are a sustainable, feasible, engaging, empowering and efficient method for both monthly review of early active disease and annual review of stable disease.

Second, by methodical iterative development using a co-design approach with patients, clinical staff and key stakeholders, enabling themes, which indicate patient satisfaction and acceptability, have been used to inform service improvement. Third, the study has also identified barriers to success, established promoting factors, demonstrated cost efficiencies and provided sustainability metrics, which support the rolling out of the group clinic model. We believe that this approach can help to alleviate some of the burden of an overstretched and overstressed health-care system in the UK. Finally, recommendations for clinical staff development in relationship to teaching and facilitation have been made, firmly grounded in the feedback from the group clinic patients. The patients have already made their satisfaction with their involvement in the co-design and their positive view of the model very clear.

There is already considerable interest in this model of care, including high-profile editorials [8, 15] and a recent review article exploring a systems approach to implementation [10]. This paper provides the data to underpin this. Serious consideration is warranted to the application of this model with inflammatory arthritis and a range of other chronic diseases more widely across primary and secondary care settings in the UK and elsewhere. Clinicians, managers and health-care commissioners who wish to implement this model of care should get in touch with the corresponding author, who will connect them with an appropriate local mentor or trainer, depending on the setting and conditions proposed.

## Supplementary Material

rkaa003_Supplementary_Data
