# Supplementary material for: Mixed methods study of a new model of care for chronic disease: co-design and sustainable implementation of group consultations into clinical practice
Source: Rheumatol Adv Pract. 2020 Jan 28;4(1):rkaa003. doi: 10.1093/rap/rkaa003 (PMC7079718; doi:10.1093/rap/rkaa003)
Supplement: rkaa003_Supplementary_Data [file rkaa003_supplementary_data.zip › Supp_Data/Online Appendix.pdf]

Group Clinics for Inflammatory Arthritis: Treatment Strategy

Northumbria Healthcare NHS
NHS Foundation Trust

DISEASE ACTIVITY & TREATMENT TARGETS

Rheumatoid Arthritis Disease Activity Score (DAS28)

A score calculated using total numbers of swollen and tender joints, patient global assessment (0 - 100) and ESR/CRP

ESR & CRP are blood tests measuring level of inflammation

Score:		
	>5.1	High disease activity
	5.1-3.2	Moderate disease activity
	<3.2	Low disease activity
	<2.6	Remission

Drop in 1.2 points indicates a good response to treatment

Psoriatic Arthritis

≤2 swollen or tender joints: Low disease activity

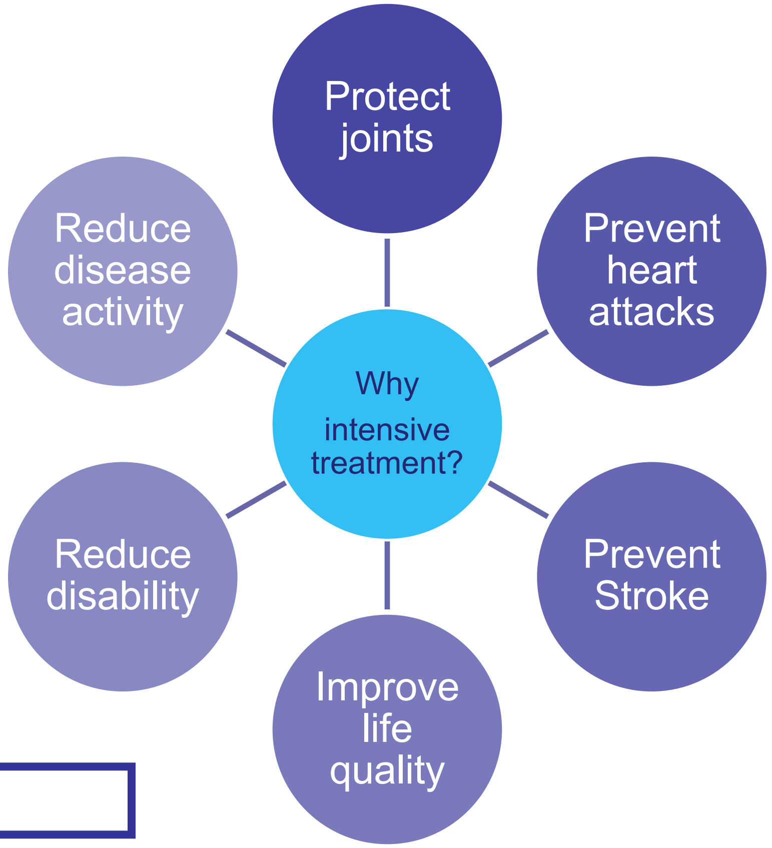

TREATMENT TO TARGET

Active disease

↓

Agree treatment target for **Low disease activity** or **Remission**

↓

Treat to target
Monthly assessment
IM Steroid if required
(as recommended from TICORA/TICOPA studies)

↓

- Target Achieved → Annual review
- Target Not achieved → Modify Therapy → (back to Treat to target)

DISEASE MODIFYING DRUG ESCALATION

Step 1
Methotrexate (MTX)
+/- at least 1 other DMARD, commonly triple therapy: MTX/SLZ/HCQ
+/- short term steroid (usually IM injection)

Step 2
If not already on, triple therapy with DMARDs:
MTX **+** Sulfasalazine **+** Hydroxychloroquine
or MTX **+** Leflunomide

Step 3
Poor response after trial of 2 DMARDs and DAS28 >5.1
Consider "Biologics" (Adalimumab, Etanercept, Infliximab) + MTX

Step 4
Inadequate response or intolerance:
Rituximab + MTX: or other biologic options e.g. Abatacept/Tocilizumab

USEFUL RESOURCES FOR PATIENTS ON THIS TOPIC

Versus Arthritis website: www.arthritisresearchuk.org
(merger of Arthritis Research UK & Arthritis Care)

TICORA Study: Grigor et al. 2004 *Lancet*.364:263-9 thelancet.com

TICOPA Study: Coates et al. 2015 *Lancet*.386: 2489-98 thelancet.com

TACIT Study: Scott et al. 2015 British Medical Journal 350:h1046 bmj.com

National Guideline (NICE NG100): www.nice.org.uk/ng100
