# Supplementary material for: Mixed methods study of a new model of care for chronic disease: co-design and sustainable implementation of group consultations into clinical practice
Source: Rheumatol Adv Pract. 2020 Jan 28;4(1):rkaa003. doi: 10.1093/rap/rkaa003 (PMC7079718; doi:10.1093/rap/rkaa003)
Supplement: rkaa003_Supplementary_Data [file rkaa003_supplementary_data.zip › Supp_Data/SUPPLEMENTARY_MATERIA5_043 1.docx]

**SUPPLEMENTARY MATERIAL**

**Figure legend**

**Supplementary Figure S1. Group Clinic Educational Poster**
